# Effect of long soaking in Kawa Daun beverage on discoloration of nanofilled composite resin

**DOI:** 10.2340/biid.v13.45575

**Published:** 2026-04-28

**Authors:** Deli Mona, Syifa Mahdiyah

**Affiliations:** Department of Dentistry, Andalas University, Padang, Indonesia

**Keywords:** Nanofilled composite resin, leaf kawa, color change, spectrophotometer

## Abstract

**Objective:**

This study investigated the effect of prolonged soaking in the Kawa Daun beverage on the discoloration of nanofilled composite resin.

**Materials and methods:**

Thirty disk-shaped specimens (6 mm diameter, 2 mm thickness) of nanofilled composite resin were prepared and divided into six groups. The samples were soaked in Kawa Daun solution, Robusta coffee solution, and artificial saliva for 6 and 18 hours at 37°C. Color measurements were taken before and after soaking using a Colorflex EZ spectrophotometer, and ΔE values were calculated. Data were analyzed using repeated-measures analysis of variance and post-hoc tests (*p* < 0.05).

**Results:**

Significant color changes were observed among the groups (*p* < 0.001). Soaking in Kawa Daun and Robusta coffee solutions produced significantly greater discoloration than artificial saliva, with increased ΔE values corresponding to longer soaking times.

**Conclusion:**

Prolonged exposure to Kawa Daun beverage caused measurable discoloration of nanofilled composite resin, likely due to pigment and acid content that degrade the resin matrix. These findings highlight the importance of patient education regarding the esthetic effects of traditional acidic and pigmented beverages on dental restorations.

**KEY MESSAGES**

1. The habit of consuming colored and acidic drinks over a long period may cause discoloration of composite resin restorations.

## Introduction

Dental restorations reconstruct lost parts of a tooth and help regain its function and appearance by utilizing restorative materials [1]. Due to the restoration frequently being noticeable, the choice of materials should focus on visual compatibility (color, transparency, texture) and lasting esthetic durability [2]. Composite resin serves as a widely used option for restorative purposes [3].

Composite resin is widely used because it can closely match tooth color [4]. Nanotechnology has led to nanocomposites intended to improve wear performance, surface smoothness, and mechanical properties through changes in filler characteristics, including particle size and filler loading, with corresponding improvements in polishability [5]. Nanofilled composite resin is one such material [1].

Nanofilled composite resin has been associated with smoother surfaces, reduced wear, and lower shrinkage while maintaining acceptable esthetics [6]. These outcomes are commonly linked to the nanomer–nanocluster filler system, which improves packing and reduces gaps within the resin matrix, strengthening physical performance. A key physical property in this context is color stability [7].

The color stability of composite resins is essential since the composite may undergo color changes at the expense of color stability. The change in color is in part the result of time-dependent phenomena and may indicate the loss of stability [8]. The color stability of composite resin is the result of intrinsic and extrinsic factors. Intrinsic factors are related to the physicochemical changes in the most internal layers of the composite resin. Extrinsic factors, such as certain pigments found in food and beverages, are capable of affecting the long-term color stability of composite resin. Food and beverages that contain color pigments that may permeate the resin matrix, which has water-absorbing properties [8]. The extrinsic factors that are likely to contribute to the changes in color of the composite resin may also include the acid molecules contained in the food and/or beverages [9].

Several studies have quantified the discoloration caused by conventional beverages and found that prolonged exposure to coffee drinks significantly increased color change in composite materials, largely due to chromogenic pigments binding to the resin surface. Similarly, the potential of hydrogen (pH) and chemical composition of staining solutions have been linked to microstructural changes in resin matrices, where acidic environments accelerate surface softening and pigment penetration [10]. Yet, the majority of these investigations have focused on widely consumed beverages in global contexts, leaving less common regional drinks underexplored. One of these drinks is Kawa Daun.

Kawa Daun is a beverage from West Sumatra made by steeping dried coffee leaves in water, yielding a drink darker than tea. The practice of consuming beverages made from coffee leaves has also been reported in Ethiopia, Jamaica, India, South Sudan, and even Indonesia [11]. Kawa Daun contains caffeine, histidine, sucrose, tannins, trigonelline, chlorogenic acid, rutin, and mangiferin [12]. Kawa Daun has been noted to help with health issues due to bioactive compounds that help in managing diabetes, providing antioxidant effects, and lowering cholesterol levels [11]. Kawa Daun also has a lower caffeine and chlorogenic acid content than coffee. Studies indicate that the chlorogenic acid may be responsible for the breakdown of polymer compounds in the composite resin, which may influence discoloration in nanofilled composite resin [10]. Despite this knowledge, there are currently no published data addressing how Kawa Daun affects nanofilled composite resins, especially given its unique composition and lower but still significant caffeine content.

This absence of data highlights a clear research gap. While the staining effects of common beverages like coffee and tea have been widely documented, the influence of traditional herbal or leaf-based drinks like Kawa Daun on the color stability of advanced restorative materials remains insufficiently explored. Understanding this relationship is essential because patients in regions where Kawa Daun is consumed may exhibit discoloration of restorative materials differently from those exposed to conventional beverages. Moreover, this knowledge could inform material selection for different dietary environments and support the development of improved composite resins with better color retention under diverse exposure conditions.

Therefore, this study evaluated the effect of prolonged soaking in Kawa Daun beverage as detailed in Table 1 on the discoloration of the nanofilled composite resin. The hypothesis proposed was that increasing the soaking duration in Kawa Daun results in more pronounced color changes, suggesting that chemical interactions between the beverage’s compounds and the resin matrix may compromise the aesthetic longevity of the restoration.

**Table 1:** Description of treatment groups.

Treatment groups	Company name	Soaking time	Temperature	pH
Kawa Daun Solution	Coffee Nicky, Kab. Tanah Datar, Indonesia	6 hours and 18 hours	37°C	6.0
Coffee Solution	Coffee Nicky, Kab. Tanah Datar, Indonesia	6 hours and 18 hours	37°C	5.0
Artificial Saliva	Material Chemistry Laboratory, Department of Chemistry, Faculty of Mathematics and Natural Sciences, Andalas University	6 hours and 18 hours	37°C	7.0

pH: Potential of hydrogen.

## Methods

### Manufacturing of composite resin samples

This research was a quantitative analytical study with a true experimental laboratory research design, utilizing a pretest–posttest control group design classification. Using a plastic filling instrument, Palfique LX5 (Tokuyama Dental Co., Japan) nanofilled composite resin was inserted into a mold made of lightweight steel (diameter = 6 mm, thickness = 2 mm). Celluloid strips were then placed above and below the composite resin mold to compress and level the composite resin samples.

The nanofilled composite resin was then light cured using a Woodpecker light-emitting diode (LED). B light curing for 20 seconds. The curing was performed on the upper side of the samples at a distance of approximately 1 mm from the surface. The sample was removed from the mold and then using an enhanced bur, applying consistent vertical pressure and direction during polishing, with 3–5 polishing strokes [7]. The polished samples were placed in a desiccator at room temperature 25°C.

This research used Kawa Daun solution as the experiment group, Robusta coffee solution as the positive control group, and artificial saliva as the negative control group. Both the Kawa Daun and coffee solutions were derived from Robusta coffee. The preparation of the Kawa Daun and coffee solutions involved using 15 grams of dried Robusta coffee leaves for the Kawa Daun solution and 15 grams of Robusta coffee powder for the coffee solution. These were then dissolved in 200 mL of water, boiled simultaneously for 10 minutes, and subsequently filtered [13]. The artificial saliva was obtained from the chemical material laboratory at Andalas University [14].

Determination of the sample size was conducted using the formula from Federer, which states [15]:

(*t* - 1)(*r* - 1) ≥15

The number of samples required was four samples for each treatment group. To account for the potential loss of experimental units or dropouts, a correction was made using the formula $N=\frac{n}{(1-f)'}$, where *f* represents the proportion of experimental units expected to drop out. Thus, the total number of samples required for each group was five samples. A power analysis was performed using G*Power software version 3.1 to determine the minimum required sample size with a significance level of α = 0.05, an effect size of 0.4, and a statistical power of 0.8 (80%). Based on the results of this analysis, a total of five samples per group satisfied the statistical power requirement to detect significant differences in color change among the treatment groups [16]. The samples were divided into six groups (group 1: composite resin samples soaked in Kawa Daun solution for 6 hours; group 2: composite resin samples soaked in Kawa Daun solution for 18 hours; group 3: composite resin samples soaked in artificial saliva for 6 hours; group 4: composite resin samples soaked in artificial saliva for 18 hours; group 5: composite resin samples soaked in Robusta coffee solution for 6 hours; and group 6: composite resin samples soaked in Robusta coffee solution for 18 hours) [9].

Each sample was measured for ΔE as pretest data before soaked. Subsequently, each group was soaked in a petri dish for the specified soaked duration. Petri dishes were filled with 10 mL of Kawa Daun solution, coffee solution, and artificial saliva until the composite samples were completely submerged and then labeled with paper codes. The samples were placed in an incubator at 37°C [7].

### Color measurement

After treatment, the samples were dried with tissue paper. They were then placed in the Colorflex EZ Spectrophotometer connected to a computer. The start button was pressed, and after several seconds of processing, the sample values were obtained. The resulting values were recorded and inserted into the color change formula to obtain the ΔE value as the final color change measurement. The ΔE value was obtained based on the difference between the coordinate parameters that were recorded before and after soaking using the following formula: CIEDE2000 [17]. Calculations were performed using the CIEDE2000 calculator from Colormine software.

The average value (∆E) and standard deviation (SD) were calculated, followed by statistical analysis using Statistical Product and Service Solutions (SPSS) software (version 27; IBM Corp., Armonk, NY, USA). The Shapiro-Wilk test and Levene’s test were employed to evaluate normality and homogeneity, which served as prerequisites for conducting a repeated-measures analysis of variance (ANOVA). The data were regarded as normally distributed and homogeneous when the *p* > 0.05. Repeated-measures ANOVA was used to compare mean ∆E values across groups. If the ANOVA indicated significant differences, post-hoc tests were conducted for pair-wise comparisons. A significance level of *p* < 0.05 was considered statistically significant.

## Results

The results are presented in Table 2 as ΔE values.

**Table 2:** Mean color changes in nanofilled composite resin after soaking according to treatment group.

Treatment group	*n*	Color change of nanofiller composite resin		
		Mean ± SD	Max	Min
Group 1	5	0.49 ± 0.08^b^	0.63	0.41
Group 2	5	1.31 ± 0.05^d^	1.37	1.23
Group 3	5	0.18 ± 0.08^a^	0.33	0.12
Group 4	5	0.19 ± 0.05^a^	0.25	0.11
Group 5	5	0.87 ± 0.09^c^	0.95	0.75
Group 6	5	1.46 ± 0.06^d^	1.52	1.38

*Group 1: composite resin samples soaked in Kawa Daun solution for 6 hours; Group 2: composite resin samples soaked in Kawa Daun solution for 18 hours; Group 3: composite resin samples soaked in artificial saliva for 6 hours; Group 4: composite resin samples soaked in artificial saliva for 18 hours; Group 5: composite resin samples soaked in Robusta coffee solution for 6 hours; Group 6: composite resin samples soaked in Robusta coffee solution for 18 hours. Note: Values with the same superscript letters are not significantly different (*p* > 0.05), whereas values with different superscript letters are statistically significant differences (*p* < 0.05). SD: standard deviation.

The data were found to be normally distributed and homogeneous, and the repeated-measures ANOVA test then performed indicated a statistically significant difference in color change among the groups (*p* < 0.001).

The post-hoc test found significant differences among the groups, except between group 3 and group 4 (composite resin samples soaked in artificial saliva for both 6 and 18 hours) and between group 2 and group 6 (composite resin samples soaked in Kawa Daun solution for 18 hours and Robusta coffee solution for 18 hours).

## Discussion

The results of this study supported the proposed hypothesis, indicating that longer soaking durations in Kawa Daun beverage caused greater color changes in nanofilled composite resin. The degree of discoloration increased with the duration of soaking, suggesting that the chemical composition of Kawa Daun influences the color stability of the composite material. This outcome aligns with the hypothesis that extended exposure to the beverage’s compounds promotes greater pigment absorption and chemical interaction with the resin matrix.

Pigments from foods or beverages that act as extrinsic staining agents may cause color changes in composite resin. High pigment concentrations, such as those found in Kawa Daun, which contains tannin pigments with an average value of 4.29% [18]. This can increase the likelihood of composite resin discolorotion [19].

The extrinsic factors of composite resin discoloration can also occur due to plaque accumulation, acid molecules in food or beverages, and depend on the degree of polymerization of the resin matrix [20]. Acid molecules in beverages are absorbed by the resin matrix; thus, the higher the absorption of acid molecules, the faster the degradation of the composite resin surface [19]. Kawa Daun (coffee leaf tea) contains lower chlorogenic acid than coffee (mean 2.1%), yet it may still cause discoloration, consistent with Sirang et al., who reported that chlorogenic acid in coffee beverages can contribute to color changes in composite resin [21, 22].

Hydrogen (H^+^) ions in food and beverage acid molecules can bind to carboxylate (COO^-^) ions in the CO_2_CH_3_ group of the polymer, causing it to break down into carboxylic acid (COOH) and methyl (CH_3_), which results in the breaking of double bonds in dimethacrylate compounds [9]. Research by Chumairo et al. indicated that longer sample soaking periods in coffee resulted in darker sample coloration [10]. This finding aligns with the results of the present study, which indicates that samples soaked for 18 hours exhibit a darker color compared to those soaked for 6 hours in both Kawa Daun solution and Robusta coffee solution. However, samples soaked in Robusta coffee solution demonstrate a greater darkness than those soaked in Kawa Daun solution, likely due to the naturally darker color of the Robusta coffee solution. Meanwhile, no significant color change was observed in samples soaked in artificial saliva or either 6 hours or 18 hours.

Water absorption can contribute to color change in composite resin [23]. In our testing of a single nanofilled composite, uptake occurs because hydrophilic monomers such as Bisphenol A-Glycidyl Methacrylate (Bis-GMA) and triethylene glyol dimethacrylate (TEGDMA) attract polar water molecules via hydroxyl/ethoxy groups and hydrogen bonding [24]. Continued absorption can hydrolyze silane bonds at the filler-matrix interface (Si-O-Si [Siloxane] → Si-OH [Silanol]), weakening the material, promoting water diffusion, and leading to hygroscopic [24, 25].

This study contributes valuable insight by examining a traditional and culturally relevant beverage, Kawa Daun, which has not been previously studied in relation to restorative material discoloration. The strength of this research lies in its novelty and its relevance to local dietary habits, providing foundational data that can inform clinical recommendations for patients whose consumption patterns differ from those typically evaluated in international literature. Additionally, the study design employed controlled conditions that allowed systematic observation of discoloration trends over time, thereby clarifying the proportional relationship between soaking duration and pigment infiltration in nanofilled composite resin.

Despite these contributions, several limitations should be acknowledged. First, this investigation was conducted under in vitro conditions, which do not fully reflect the complexity of the oral environment. Factors such as salivary flow, enzymatic activity, temperature variation, and mechanical abrasion from mastication were not simulated. These conditions may significantly influence the discoloration behavior of resin composites in vivo. Second, the study was limited to a single type of nanofilled composite resin and one beverage solution, which restricts the generalizability of the findings to other materials or staining agents. Variations in filler particle size, matrix composition, and finishing techniques could yield different results under similar exposure. Third, while the color measurements captured visible changes, the study did not assess surface roughness, microhardness, or chemical degradation at the microscopic level, which could provide more detailed insight into the underlying mechanisms of discoloration.

Future research should therefore include both in vitro and in vivo analyses that more accurately represent the oral environment. Investigations comparing natural and synthetic drinks with different pH levels and pigment compositions could enhance understanding of how beverage chemistry interacts with resin matrices. In addition, exploring multiple composite systems with different filler types and testing parameters such as brushing simulation, temperature cycling, and enzyme exposure would yield a more comprehensive evaluation of color stability.

## Conclusion

These results imply that composite resin materials may be susceptible to surface deterioration and color absorption when exposed to acidic, colored conditions. The statistically significant differences in color change seen following 6 hours and 18 hours of soaking in Kawa Daun solution support this conclusion.

In practical terms, this suggests that frequent consumption of naturally tinted beverages, herbal drinks, or acidic foods may gradually compromise the esthetic integrity of composite restorations. Over time, such exposure could lead to noticeable discoloration, affecting patient satisfaction and potentially prompting premature replacement of restorations. Therefore, clinicians should emphasize preventive advice, including moderating intake of staining substances and performing immediate rinsing or brushing after consumption. Additionally, the selection of composite materials with greater color stability and improved surface resistance should be prioritized for restorations in esthetically demanding areas. By integrating these findings into clinical decision-making, dental practitioners can enhance the longevity and visual quality of composite restorations, thereby improving overall treatment outcomes.

## Data Availability

The data that support the findings of this study are available from the corresponding author upon reasonable request.
